# Characteristics of Aflatoxin B_1_ Degradation by *Stenotrophomonas acidaminiphila* and It’s Combination with Black Soldier Fly Larvae

**DOI:** 10.3390/life13010234

**Published:** 2023-01-14

**Authors:** Jianghua Suo, Tingting Liang, Haixu Zhang, Kun Liu, Xinfeng Li, Kun Xu, Jianlai Guo, Qiujiang Luo, Sen Yang

**Affiliations:** 1 College of Animal Science, Xinjiang Agricultural University, Urumqi 830052, China; 2 College of Food and Bioengineering, Henan University of Animal Husbandry and Economy, Zhengzhou 450060, China; 3 College of Life Sciences, Henan Agricultural University, Zhengzhou 450002, China; 4 Henan Key Laboratory of Innovation and Utilization of Unconventional Feed Resources, Henan University of Animal Husbandry and Economy, Zhengzhou 450060, China; 5 Animal and Aquatic Products Inspection &Testing Technologies, Henan Institute of Agricultural, Zhengzhou 451450, China

**Keywords:** alatoxins, biodegradation, black soldier fly larvae, *Stenotrophomonas acidaminiphila*

## Abstract

**Simple Summary:**

Aflatoxin B_1_ (AFB_1_), one of the most hazardous mycotoxins commonly present in food and feed, causes great loss in livestock farming and severe safety risks to human health. In this paper, benefiting from using a sterile black soldier fly larvae (BSFL) system, we found that the ability of BSFL to degrade AFB_1_ was greatly reduced in the absence of gut microbiota, which indicated the important role of gut microbiota in AFB_1_ degradation. Furthermore, twenty-five AFB_1_-degrading bacteria were isolated from BSFL gut, and *S. acidaminiphila* A2 achieved the highest AFB_1_ degradation, by 94%. When *S. acidaminiphila* A2 was re-inoculated to BSFL, the detrimental effect of AFB_1_ on the growth performance of BSFL was alleviated, and complete AFB_1_ degradation in peanut meal was obtained. The present study may provide a strategy to degrade AFB_1_ in feedstuff through bioconversion with BSFL in combination with gut-originated AFB_1_-degrading bacteria, while providing a sustainable insect protein and fat source to animals.

**Abstract:**

Aflatoxin B_1_ (AFB_1_) is a common mycotoxin contaminant in cereals that causes severe economic losses and serious risks to the health of humans and animals. In this paper, we investigated the characteristics of AFB_1_ degradation by black soldier fly larvae (BSFL) combined with commensal intestinal microorganisms. Germ-free BSFL and non-sterile BSFL were reared on peanut meal spiked with AFB_1_ for 10 days. The result showed that germ-free BSFL and non-sterile BSFL could achieve 31.71% and 88.72% AFB_1_ degradation, respectively, which indicated the important role of larvae gut microbiota in AFB_1_ degradation. Furthermore, twenty-five AFB_1_-degrading bacteria were isolated from BSFL gut, and *S. acidaminiphila* A2 achieved the highest AFB_1_ degradation, by 94%. When *S. acidaminiphila* A2 was re-inoculated to BSFL, the detrimental effect of AFB_1_ on the growth performance of BSFL was alleviated, and complete AFB_1_ degradation in peanut meal was obtained. In conclusion, the present study may provide a strategy to degrade AFB_1_ in feedstuff through bioconversion with BSFL in combination with gut-originated AFB_1_-degrading bacteria, while providing a sustainable insect protein and fat source to animals.

## 1. Introduction

Aflatoxins (AFs) are a group of toxic secondary metabolites produced by the *Aspergillus* species, notably *Aspergillus flavus* and *Aspergillus parasiticus*, that frequently contaminate corn, rice, peanuts, nuts, oilseeds and their derived products [1]. Among the twenty AFs identified so far, aflatoxin B_1_ (AFB_1_) is the most harmful and is responsible for more than 75% of all AFs contamination in food and feed [2]. Although many prevention strategies have been adopted in the field and during storage, AFB_1_ contamination in food and feed occurs worldwide [3]. AFB_1_-related health problems tend to be the most severe in developing countries, and more than 4.5 billion people are chronically exposed owing to the lack of regulation of AFB_1_ [4]. AFB_1_ can be activated by hepatic cytochrome P450 enzymes to produce genotoxic intermediate exo-AFB-8, 9-epoxide (AFBO), which can react with the N^7^ atom of guanine to form pro-mutagenic DNA adducts [5]. The International Agency for Research on Cancer (IARC) has classified AFB_1_ as a Group I human carcinogen [6]. The global burden of AFB_1_-driven hepatocellular carcinoma (HCC) or liver cancer is as high as 155,000 cases [7]. In addition to its genotoxic proprieties, AFB_1_ threatens the health of both humans and animals by inducing hepatotoxicity, nephrotoxicity, teratogenicity and immunotoxicity [8]. Therefore, the development of effective strategies to detoxify AFB_1_ in contaminated food and feed has attracted tremendous attention.

To date, several biological, physical, and chemical approaches have been developed for the detoxification of AFB_1_ [8]. Compared with the physical or chemical methods, biodegradation—especially microbial degradation—is usually considered to be low-cost and friendly to the environment, and can transform AFB_1_ into less toxic or non-toxic metabolites under mild conditions, retaining the palatability and nutritive quality of food commodities [9]. Several studies have reported many bacterial strains with AFB_1_-degrading ability, such as *Pseudomonas putida* 1274 [10], *Streptomyces aureofaciens* ATCC10762 [11], *Rhodococcus globerulus* AK36 [12] and *Bacillus subtilis* ANSB060 [13].

As the most abundant number of animals, insects play various roles in human life [14]. In recent years, extensive attention has focused on black soldier fly larvae (BSFL), *Hermetia illucens* (L.) (Diptera: Stratiomyidae) for the treatment of organic pollutants due to their large food intake rate and high conversion efficiency [15]. BSFL are rich in protein and have a well-balanced essential amino acid profile similar to the amino acids of fishmeal, and thus can provide high-value feedstuff [16]. Moreover, BSFL also have a high fat content, the composition of which will vary with feeding materials [17]. They can partially replace soybean meal, fish meal and fish oil in the fields of animal husbandry and aquaculture [18,19]. Previous studies have reported that BSFL could degrade aflatoxins without accumulation in the body of harvested larvae [20,21]. BSFL carry abundant microbial resources, and their symbiotic and intestinal microbes are of great significance to the life activities of the host. Despite the contribution of BSFL gut microbiota, the roles of microbiota in BSFL-mediated aflatoxins degradation remain poorly understood. In the current study, we investigated the feasibility and capability of BSFL-mediated AFB_1_ degradation in contaminated peanut meal. The AFB_1_-degrading bacterial strains were screened and isolated to provide direct evidence on the contribution of the BSFL-associated microbiota to the degradation. Moreover, we further explored the impact of the AFB_1_-degrading isolate on the degradation performance and growth performance, as well as the nutrient compositions, of BFSL. The findings will provide a novel strategy to degrade AFB_1_ in feedstuff with BFSL combined with commensal intestinal microorganisms.

## 2. Materials and Methods

### 2.1. Chemicals and Medium

AFB_1_ standard was purchased from Sigma (Shanghai, China), was dissolved in methanol (1 mg mL^−1^) to prepare a stock solution and was stored in the dark at −20 °C. Methanol (HPLC grade) was obtained from ROE scientific incorporation (Newark, NJ, USA). Water was purified by a Milli-Q Water System (Millipore Corporation, New York, NY, USA). All other reagents were of analytical grade and were obtained from Sinopharm Chemical Reagent Co., Ltd. (Beijing, China). Luria–Bertani (LB) medium contained the following constituents: 5 g L^−1^ yeast extract, 10 g L^−1^ NaCl and 10 g L^−1^ tryptone. LB medium was autoclaved at 121 °C for 15 min. The solid medium was added to 15 g L^−1^ agar based on the liquid medium.

### 2.2. Germ-Free Intestinal BSFL Model Construction

Germ-free BSFL was constructed as described in our previous study [22]. Fresh BSFL eggs provided by the BSFL Culturing Center of Henan Agricultural University were placed in a sterile centrifuge tube containing 1 mL of 2.7% NaClO solution and 1 mL of Sporgon (Beijing Mingyangkehua Bio-Technology, Beijing, China) and were shaken gently to disperse the eggs thoroughly. The disinfectant was decanted and the eggs were rinsed with sterile water three times. Disinfected eggs were inoculated into the brain heart infusion (BHI) medium and were incubated at 37 °C for 24 h. The culture was subsequently spread on the LB agar plates to verify the disinfection effect.

### 2.3. Degradation of AFB_1_ in Peanut Meal by BSFL

Germ-free BSFL and non-sterile BSFL were reared on autoclaved wheat bran at 30 °C with 70% moisture content until 3rd instar larvae. Subsequently, twenty BSFL were inoculated into 9.0 g autoclaved peanut meal containing 100 ng g^−1^ AFB_1_. The BSFL were continuously reared for 10 days at 30 °C with 70% relative humidity. Three layers of gauze were added to the outside the incubator to maintain humidity and prevent larvae from creeping out. The AFB_1_-contaminated peanut meal without BSFL treatment was also put in the same condition to serve as the control. The BSFL and frass were separated by manual sieving with a 3 mm mesh and were dried at 75 °C for subsequent AFB_1_ content analysis. The frass refers to the feces of the larvae and the residual substances of the feed. The AFB_1_ content of all the samples was determined with high-performance liquid chromatography (HPLC) as described in Section 2.8. The AFB_1_ degradation rate was calculated as: DR = (M1 − M2)/M1 × 100%, where M1 was the total AFB_1_ content in the peanut meal, and M2 was the total AFB_1_ content in the frass.

### 2.4. Screening and Identification of AFB_1_-Degrading Bacteria from BSFL Gut

Larvae from AFB_1_-contaminated peanut meal were surfaced cleaned with 75% alcohol and sterile 1% NaCIO solution, and sterile water. The intestines were dissected and washed. One gram of intestine sample was added to 10 mL of peptone water (0.1% *w*/*v*), mixed well by vortex and agitated for 30 min. The sample was diluted from 10^−1^ to 10^−7^ with sterile distilled water, and 100 μL of each dilution was spread on LB agar medium containing 1 μg mL^−1^ AFB_1_ at 37 °C for 48 h. Single colonies were streaked on the same medium in different petri dishes to purify each isolate. Subsequently, the bacterial isolates were cultured in 50 mL of LB medium at 37 °C for 24 h and then tested for AFB_1_ degradation capacity. An aliquot of 990 μL of bacterial culture was mixed with 10 μL of 10 μg mL^−1^ AFB_1_ standard stock solution, and incubated at 37 °C for 48 h. In the control group, the bacterial culture was replaced with a sterile LB medium. The residual AFB_1_ was extracted and quantified with high-performance liquid chromatography (HPLC). The AFB_1_ degradation rate was calculated using the following equation: DR = (1 − C_T_/C_C_) × 100%, where DR was the degradation rate and CT and CC were the AFB_1_ concentration of experimental treatments and the control group, respectively.

Taxonomic characterization of the screened AFB_1_-degrading bacteria was performed by sequence analysis of the 16S rRNA gene. The genomic DNA was extracted using a Rapid Bacteria Genomic DNA Isolation Kit (Sangon Biotech, Shanghai, China). The 16S rRNA gene was amplified with universal primer 27F (5′-AGAGTTTGATCCTGGCTCAG-3′) and 1492R (5′-TACGACTTAACCCCAATCGC-3′). The obtained sequence was analyzed using a BLAST search in the NCBI database (http://blast.ncbi.nlm.nih.gov/blast, accessed on 3 March 2020), and was aligned using the Clustal W program. The phylogenetic tree was constructed by MEGA 7.0 according to the neighbor-joining algorithms with 1000 bootstrapping.

### 2.5. Characterization of the AFB_1_ Degradation Ability of Stenotrophomonas acidaminiphila Strain A2

To determine the functional detoxification component, we analyzed the effects of the culture supernatant, cell pellets and cell fragments of the strain A2 on the AFB_1_ removal ability. The bacterial culture was centrifuged at 12,000× *g* for 10 min. The culture supernatant and cell pellets were separated and then the supernatant was filtered with a 0.22 μm filter before use. The pellets were washed twice in PBS and then given an equal volume of PBS. A portion of cell suspension was sonicated on ice for 1 min and centrifuged at 12,000× *g* for 15 min at 4 °C to obtain cell fragments. The bacterial culture, culture supernatant, cell pellets and cell fragments were individually incubated with AFB_1_ at 37 °C for 24 h. The sterile LB medium with AFB_1_ was incubated for the same duration of time as a control. The experiment was conducted in triplicate. The effects of incubation time, pH, temperature, metal ions and AFB_1_ content on AFB_1_ removal by the strain A2 culture supernatant were further evaluated. The bacterial culture supernatant was incubated with AFB_1_ for 8, 12, 16, 20, 24, 28, 32 and 36 h. The effect of pH on AFB_1_ degradation was studied by adjusting the pH of bacterial culture supernatant to 5.0, 6.0, 7.0, 8.0 and 9.0 with 1 M HCI or 1 M NaOH and then incubating the samples at 37 °C for 24 h. The effect of temperature on AFB_1_ degradation was studied by incubating the bacterial culture supernatant with AFB_1_ at 20, 30, 40, 50, 60, 70, 80, 90 and 100 °C for 24 h. The effect of metal ions on AFB_1_ degradation was explored by adding Na^+^, Cu^2+^, Mn^2+^, Mg^2+^, Ca^2+^, Zn^2+^ and Fe^3+^ to the bacterial culture supernatant at the final concentration of 1 mM, which was then incubated with AFB_1_ at 37 °C for 24 h. Moreover, the AFB_1_ degradation capacity of the bacterial culture supernatant was investigated at different initial AFB_1_ concentrations (100, 200, 300, 400 and 500 ng mL^−1^).

### 2.6. The Influence of Stenotrophomonas acidaminiphila Strain A2 on the Growth Performance and AFB_1_ Degradation Capacity of BSFL

Non-sterile BSFL were first reared on wheat bran until the 3rd instar larvae, and they were then divided into three groups. For the control group, twenty non-sterile BSFL and 9.0 g of autoclaved peanut meal were added. For the AFB_1_ group, twenty non-sterile BSFL were inoculated into 9.0 g of autoclaved peanut meal containing 100 ng g^−1^ AFB_1_. For the AFB_1_+A2 group, twenty non-sterile BSFL and 1 mL of *Stenotrophomonas acidaminiphila* strain A2 culture suspension (OD600 = 1.0) were added to 9.0 g of autoclaved peanut meal containing 100 ng g^−1^AFB_1_. Moreover, 1 mL of *Stenotrophomonas acidaminiphila* strain A2 culture suspension (OD600 = 1.0) was inoculated into 9.0 g of autoclaved peanut meal containing 100 ng g^−1^ AFB_1_ without BSFL. The experiments were performed for 10 days in an incubator at 37 °C with 70% relative humidity. The survival rate, feed consumption rate and feed conversion rate of BSFL were determined as follows. The survival rate of BSFL (%) = (N1/N2) × 100%, where N1 and N2 were the BSFL survival numbers before and after rearing, respectively. Feed consumption rate of BSFL (%) = (W1 – W2)/W1 × 100%, where W1 was the weight of dry matter of peanut meal, and W2 was the weight of dry matter of frass. Feed conversion rate of BSFL (%) = (W3 − W4)/W1 × 100%, where W1 was the weight of dry matter of peanut meal, and W3 and W4 were the dry weight of BSFL before and after rearing, respectively. The AFB_1_ degradation rate was determined as described in Section 2.3.

Dried BSFL samples were ground into homogenous powder for the measurement of nutritional compositions. The crude protein content was measured using hte Kjeldahl method based on GB 5009.5-2016 [23]; crude fat content was determined by Soxhlet Extraction based on GB 5009.6-2016 [24]; crude ash content was determined using the burning weighing method based on GB 5009.4-2016 [25]. The amino acid contents of BSFL were determined by liquid chromatography (LC) based on the method described by GB 5009.124-2016 [26].

### 2.7. Quantification of AFB_1_ by HPLC

AFB_1_ quantification was carried out by a high-performance liquid chromatography (HPLC) coupled with a fluorescence detector (Shimadzu, Tokyo, Japan). Detection conditions were: excitation at 360 nm and emission at 440 nm. A Diamonsil^®^ C18 reverse phase column (5 μm, 4.6 × 150 mm) was used for separation with the mobile phase consisting of water: methanol (45:55, *v*/*v*) at a flow rate of 1 mL min^−1^. The sample injection volume was 10 µL. The AFB_1_ standard samples were prepared in methanol with the concentration gradients (0, 6.25, 12.5, 25, 50, 75 and 100 ng mL^−1^) to establish the standard curve for AFB_1_ concentration calculation.

### 2.8. Statistical Analysis

All experiments were performed with three replications. Student’s t-test was used to determine the statistical significance of AFB_1_ degradation rate between GF-BSFL and NS-BSFL groups. A one-way analysis of variance (ANOVA) with Tukey’s multiple comparison test was performed to compare survival rate, feed consumption rate, body length, average dry weight and feed conversion rate among CON, AFB_1_ and AFB_1_+A2 groups. The difference was regarded as statistically significant when *p* < 0.05.

## 3. Results

### 3.1. Comparison of AFB_1_ Degradation Ability of Germ-Free BSFL and Non-Sterile BSFL

The AFB_1_ was not detected in the body of germ-free BSFL and non-sterile BSFL, which suggested that BSFL rapidly excreted or catabolized AFB_1_ after ingestion. As shown in Table 1, the frass weight, AFB_1_ concentration in frass and total residual AFB_1_ content in frass in non-sterile BSFL treatment were significantly lower than those in the germ-free BSFL treatment. The AFB_1_ degradation rate reached 31.71% and 88.72%, respectively, after treatment of germ-free BSFL and non-sterile BSFL.

### 3.2. Screening and Identification of AFB_1_-Degrading Bacteria from BSFL Gut

**Figure 1:**
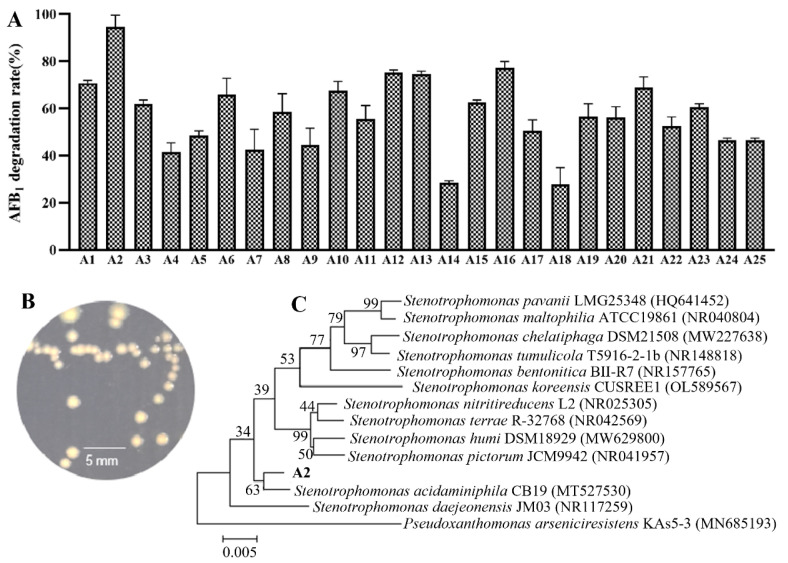
Isolation and identification of AFB_1_-degrading bacterial strains from BSFL gut. (**A**) The AFB_1_ degradation rate by culture medium of different strains. Values are means with their standard errors represented by vertical bars (*n* = 3). (**B**) The colony morphology of the strain A2. (**C**) The phylogenetic tree of the strain A2.

Individual isolates tested for AFB_1_ degradation from the larval gut would provide direct evidence for AFB_1_ degradation by gut microbiota. Two hundred and sixty eight bacteria were isolated from BSFL gut, and twenty-five of them displayed AFB_1_-degrading ability with a degradation percentage ranging from 28% to 94% (Figure 1A). The colony of the strain A2, which displayed the highest AFB_1_ degradation rate, was faint yellow with non-smooth edges on LB agar (Figure 1B). Phylogenetic analysis indicated that the strain A2 belonged to the genus *Stenotrophomonas* in the phylogenetic clade of *Stenotrophomonas acidaminiphila* (Figure 1C). The 16S rRNA gene of the strain A2 was 99.36% similar to that of *S. acidaminiphila* CB19. The taxonomic identification of ten other bacterial strains capable of degrading more than 60% of AFB_1_ is shown in Table S1.

### 3.3. Characterization of the AFB_1_ Degradation Ability of S. acidaminiphila Strain A2

**Figure 2:**
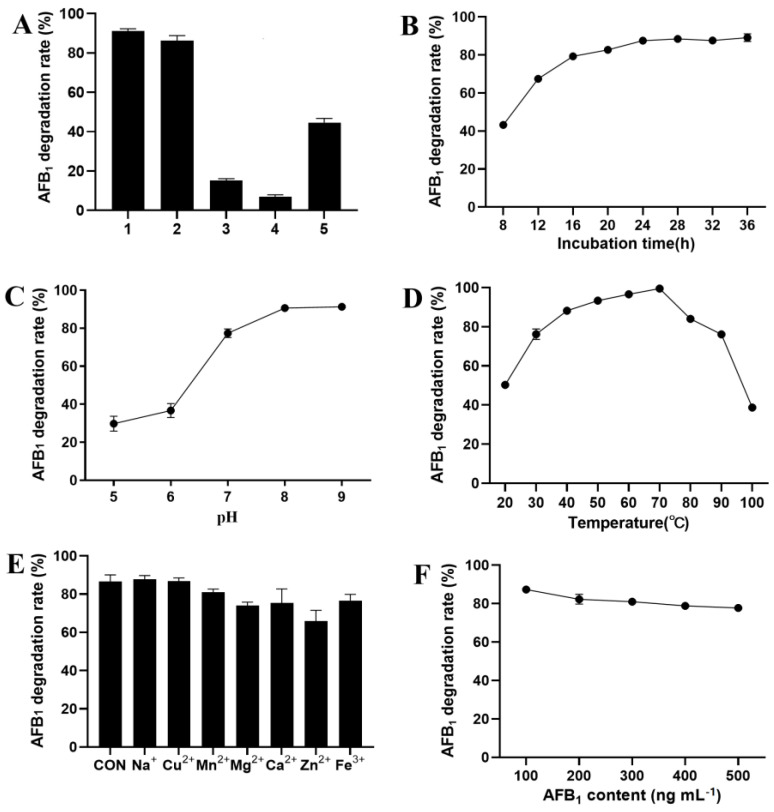
Characterization of the AFB_1_-degrading capacity of *S. acidaminiphila* A2. (**A**) The degradation of AFB_1_ by different components of the strain A2. 1, Bacterial culture; 2, Culture supernatant; 3, Cell pellets; 4, Cell fragments; 5, Culture supernatant + Proteinase K. The effects of incubation time (**B**), pH (**C**), temperature (**D**), metal ions (**E**) and substrate concentration (**F**) on AFB_1_ degradation by cell-free culture supernatant of *S. acidaminiphila* A2. Values are means with their standard errors represented by vertical bars (*n* = 3).

In order to confirm the components for the degradation of AFB_1_ by strain A2, the bacterial culture, cell-free culture supernatant, cell pellets and cell fragments were individually tested for their AFB_1_-degrading abilities. The cell-free culture supernatant, cell pellets and cell fragments displayed quite different degradation activities. The cell-free culture supernatant achieved 86% AFB_1_ degradation, while the cell pellets and cell fragments could only degrade AFB_1_ by less than 20% (Figure 2A). Thus, the major active components of the strain A2 for AFB_1_ degradation were in the cell-free culture supernatant. The proteinase K treatment could destroy the active components in culture supernatant, and resulted in a decrease of the AFB_1_ degradation rate to 44%. The AFB_1_ degradation by the strain A2 cell-free culture supernatant was time-dependent. As shown in Figure 2B, the AFB_1_ degradation rate was only 43% at 8 h, and increased gradually with the increase of incubation time up to 24 h. The degradation of AFB_1_ was sensitive to the pH value. The cell-free culture supernatant could effectively degrade AFB_1_ at neutral and alkaline pH, achieving 77% to 92% AFB_1_ degradation at pH 7.0 to 9.0 (Figure 2C). However, the percentage of AFB_1_ degradation decreased to less than 40% at pH 5.0 and 6.0. The effect of temperature on AFB_1_ degradation by the strain A2 cell-free culture supernatant is shown in Figure 2D. The AFB_1_ degradation rate ascended rapidly from 50% to 88% as the temperature rose from 20 to 40 °C, and then increased slightly to almost 100% at 70 °C. The further increase of incubation temperature led to a rapid decrease of AFB_1_ degradation rate. The effects of metal ions showed that the presence of Zn^2+^ resulted in the reduction of AFB_1_ degradation rate from 86% to 65%, while Na^+^, Cu^2+^, Mn^2+^, Mg^2+^, Ca^2+^ and Fe^3+^ showed little effect on AFB_1_ degradation (Figure 2E). Another important factor that can affect the AFB_1_ degradation rate is the substrate concentration. The result presented in Figure 2F shows that the cell-free culture supernatant was able to degrade more than 78% of AFB_1_ at an initial concentration of 100 to 500 ng mL^−1^.

### 3.4. Effect of Stenotrophomonas acidaminiphila Strain A2 on the Growth Performance of BSFL

**Figure 3:**
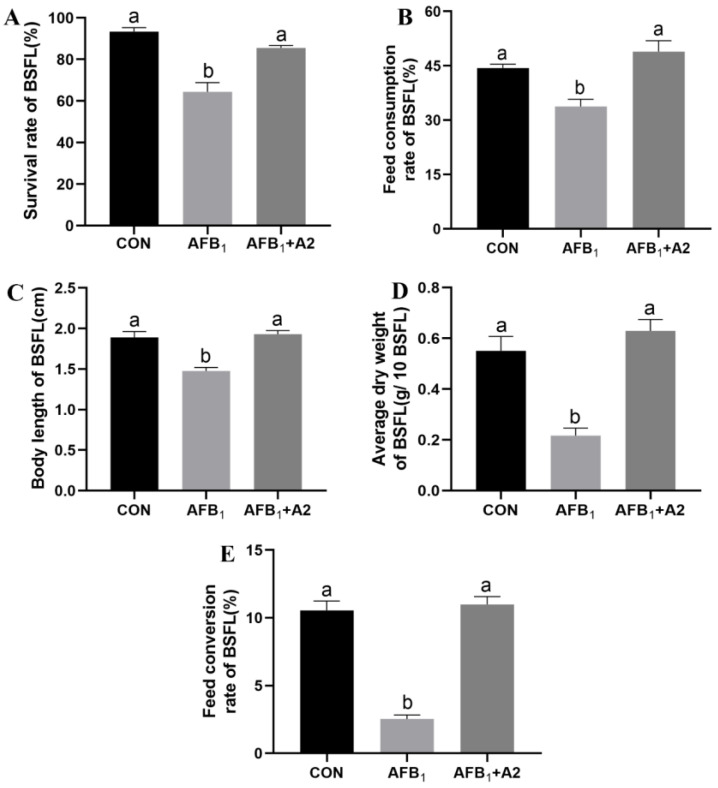
Effects of *S. acidaminiphila* A2 on the growth performance of BSFL in AFB_1_ contaminated peanut meal. (**A**) Survival rate, (**B**) Feed consumption rate, (**C**) Body length, (**D**) Average dry weight, and (**E**) Feed conversion rate. Values are means with their standard errors represented by vertical bars (*n* = 3). Values with different letters differ significantly (*p* < 0.05).

It is shown in Figure 3A that feeding the peanut meal without AFB_1_ resulted in a BSFL survival rate of 93%, indicating that the applied rearing condition was suitable to support its growth and development. However, the survival rate of BSFL reduced to 64% under exposure to AFB_1_ in the same condition. After the inoculation of *S. acidaminiphila* A2 into the AFB_1_-contaminated peanut meal, the survival rate of BSFL increased up to 86%. As presented in Figure 3B–E, the feed consumption rate, body length, average dry weight and feed conversion rate of BSFL were significantly reduced in the AFB_1_ group in comparison with the CON group, but no remarkable differences were observed between the AFB_1_+A2 group and the CON group. Thus, *S. acidaminiphila* A2 could effectively alleviate the toxicity of AFB_1_ to BSFL. In addition, the nutritional compositions of BSFL were determined (Table 2). The exposure to AFB_1_ did not significantly influence crude ash and crude protein content, but resulted in an increase of crude fat in the body of BSFL. The addition of *S. acidaminiphila* A2 in AFB_1_-contaminated peanut meal slightly decreased the crude protein content in harvested BSFL. A total of seventeen free amino acids, including eight essential amino acids, were found in BSFL. There were no significant differences in thirteen individual free amino acids among the three groups. The Proline and Alanine content in BSFL harvested in the AFB_1_ and AFB_1_+A2 groups were lowered in comparison with the CON group. The Serine and Cystine content in BSFL harvested in the AFB_1_ group were significantly reduced after the addition of *S. acidaminiphila* A2.

### 3.5. Degradation of AFB_1_ by BSFL Combined with Stenotrophomonas acidaminiphila Strain A2

**Figure 4:**
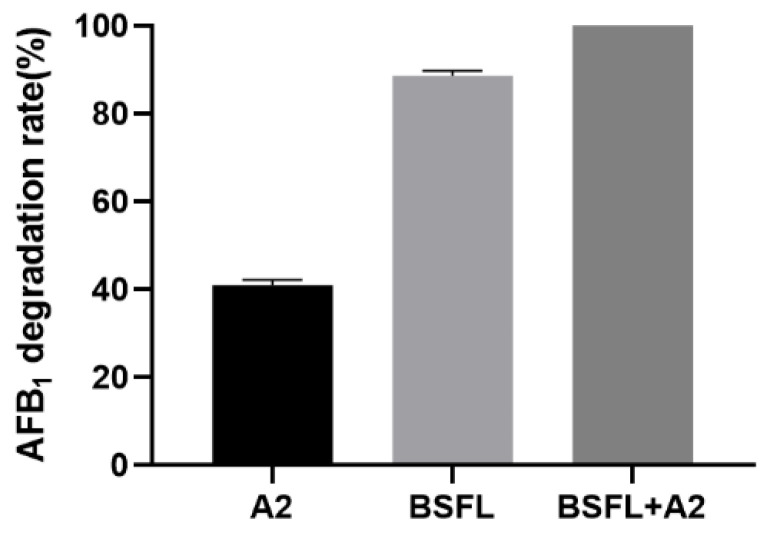
Degradation of AFB_1_ in peanut meal by BSFL in combination with *S. acidaminiphila* A2. Values are means with their standard errors represented by vertical bars (*n* = 3).

The AFB_1_ synergistic degradation between BSFL and commensal *S. acidaminiphila* A2 was explored. As shown in Figure 4, *S. acidaminiphila* A2 could degrade 41% AFB_1_ in peanut meal after 10 days, while complete AFB_1_ degradation was achieved by BSFL in combination with *S. acidaminiphila* A2.

## 4. Discussion

Among numerous saprophagous insects, BSFL have become a strikingly good candidate for bioconversion. They can effectively convert various organic wastes, such as animal manure, food waste and agricultural residues, to obtain high-quality insect biomass rich in protein and fat [16]. Previous research has shown that BSFL can biodegrade six mycotoxins (aflatoxins B1/B2/G2, deoxynivalenol, ochratoxin A, zearalenone) and three pesticides (chlorpyrifos, chlorpyrifos-methyl, pirimiphos-methyl) with no bioaccumulation in the larvae harvested [27]. Moreover, Meijer et al. [28] found that cytochrome P450 enzymes and cytoplasmic reductases were involved in the metabolic conversion of AFB_1_ in BSFL, which could transform AFB_1_ into aflatoxicol and aflatoxin P_1_. Benefiting from using a sterile BSFL system, we found that the ability of BSFL to degrade AFB_1_ was greatly reduced in the absence of microbiota, which indicated the important role of microbiota in the degradation. Indeed, insect gut microbiota can be considered an additional organ with fluidity or malleability. The larval intestinal microbiota is a crucial contributor to the BSFL nutrient metabolic process [22] and helps the host to tolerate heavy metals [29] and degrade antibiotics [30,31]. Lou et al. [30] identified two bacterial strains—*Alcaligenes faecalis* GLD-1 and *Ochrobactrum intermedium* GLD-2—capable of degrading lincomycin from BSFL gut. Yang et al. [31] found that ciprofloxacin degradation by BSFL was associated with intestinal microorganisms, and five strains that degrade ciprofloxacin were identified including *Klebsiella pneumoniae* BSFLG-CIP1, *Trichosporon asahii* BSFLG-CIP2, *Geotrichum* sp. BSFLG-CIP3, *Pichia kudriavzevii* BSFLG-CIP4 and *Proteus mirabilis* BSFLG-CIP5. In the current study, twenty-five AFB_1_-degrading bacteria were isolated from the intestine of BSFL reared on AFB_1_-contaminated peanut meal. Among them, *Stenotrophomonas acidaminiphila* strain A2 displayed the highest AFB_1_ degradation rate of 94%. Similarly, Guan et al. [32] previously obtained a *Stenotrophomonas Maltophilia* from tapir feces, which could reduce AFB_1_ by 82.5% in the liquid medium at 37 °C for 72 h. Cai et al. [33] reported that *Stenotrophomonas* sp. CW117 isolated from soils was able to degrade more than 90% of AFB_1_ at the initial concentration of 40 to 4000 μg L^−1^ within 24 h.

The active components responsible for AFB_1_ degradation were located in the culture supernatant of *S. acidaminiphila* A2. This is in agreement with the report of Cai et al. [33], who studied the AFB_1_ degradation mechanism by *Stenotrophomonas* sp. CW117 and revealed that extracellular enzymes and non-protein components were responsible for the degradation activity. In a previous study of Zhao et al. [34], a 32 kDa extracellular enzyme with AFB_1_ degradation capacity was purified from *Myxococcus fulvus*. Guo et al. [35] reported that the spore CotA laccase from *Bacillus licheniformis* could catalyze the C3-hydroxylation of AFB_1_, resulting in the formation of non-toxic transformation products aflatoxin Q_1_ and epi-aflatoxin Q_1_. Further study was needed to elucidate the enzymatic mechanisms for AFB_1_ degradation in *S. acidaminiphila* A2. The degradation of AFB_1_ by *S. acidaminiphila* A2 depends on factors such as incubation time, pH, temperature, metal ions and substrate concentration. The AFB_1_ degradation by the culture supernatant of *S. acidaminiphila* A2 was a relatively rapid and continuous process, with 67% AFB_1_ degraded in the first 12 h and 87% degraded after 24 h. Similar results were obtained elsewhere. Cai et al. [33] reported a 52% reduction of AFB_1_ within 6 h by a cell-free supernatant of *Stenotrophomonas* sp. CW117. *Microbacterium proteolyticum* B204 isolated from bovine feces could eliminate about 80% of AFB_1_ after a 12 h treatment [36]. The culture supernatant of *S. acidaminiphila* A2 could effectively degrade AFB_1_ under neutral and alkaline pH conditions, but gradually lost its AFB_1_ degradation capacity at acidic pH. The effect of pH on AFB_1_ degradation by a culture supernatant of *S. maltophilia* 35-3 showed a similar trend [32]. Moreover, the highest AFB_1_ degradation was achieved at pH 7 and 8 by *M. proteolyticum* B204 [36]. Regarding the effect of temperature, we found that high temperature did not inhibit the AFB_1_ degradation by a culture supernatant of *S. acidaminiphila* A2. Indeed, the degradation rate increased with the increase of temperature from 20 °C to 70 °C. Samuel et al. [37] also reported that the percentage of AFB_1_ degradation by culture supernatant of *Pseudomonas aeruginosa* N17-1 was elevated from 45% at 20 °C to 90% at 65 °C. The culture supernatant of *Trichoderma reesei* CGMCC3.5218 could achieve more than 90% AFB_1_ degradation at a temperature range from 45 °C to 90 °C [38]. These results imply that the enzymes or proteins involved in the AFB_1_ degradation are thermostable. Various metal ions were examined for their effects on the AFB_1_ degradation, and it was shown that the presence of Zn^2+^ led to a decrease of AFB_1_ degradation rate by a culture supernatant of *S. acidaminiphila* A2. The inhibitory effect of Zn^2+^ was commonly observed on AFB_1_-degrading strains including *Stenotrophomonas* sp. CW117 [33], *P. aeruginosa* N17-1 [37] and *T. reesei* CGMCC3.5218 [38].

The growth performance of BSFL in AFB_1_-contaminated peanut meal was investigated. The exposure to AFB_1_ resulted in a decrease of the survival rate and dry weight of BSFL as well as a reduction of feed consumption rate and feed conversion rate. However, Bosh et al. [20] previously found that BSFL displayed a high tolerance to AFB_1_, and their survival rate and body weight were not influenced when fed with poultry feed spiked with 0.5 mg kg ^−1^ AFB_1_. BSFL are considered a promising alternative protein and fat source for use in animal feed. Interestingly, we found that the presence of AFB_1_ in peanut meal could increase crude fat content by 2% but did not influence crude protein content in BSFL. In a recent study by Zhao et al. [39], yellow mealworms reared on AFB_1_-containing bran could provide the same quality of available larval protein and fat as those fed on bran without AFB_1_. The effect of AFB_1_ on fat metabolism in the two different insects is worth further study. The presence of *S. acidaminiphila* A2 could alleviate the detrimental effect of AFB_1_ on the growth performance of BSFL. Functional bacteria isolated from the BSFL gut have been found to benefit the growth of BSFL. Pei et al. [22] reported that *Bacillus velezensis* EEAM 10B could improve the protein synthesis process and digestive enzyme activities in BSFL, thus elevating the substance uptake and protein conversion ability of BSFL. When the intestinal ciprofloxacin-degrading isolates were re-inoculated to the sterile BSFL system, the larvae survival rate and weight were significantly increased in comparison with the sterile BSFL system [31]. Moreover, the addition of ciprofloxacin-degrading isolates could enhance the ciprofloxacin degradation efficiency of the sterile BSFL system. In our study, the AFB_1_ in peanut meal could be completely degraded by BSFL in combination with *S. acidaminiphila* A2. The metabolic pathways of AFB_1_ in BSFL and *S. acidaminiphila* A2 should be further investigated and clarified in depth. A toxicological test of the AFB_1_ degradation product is also necessary.

## 5. Conclusions

The present study indicated that AFB_1_ in peanut meal could be degraded by 31.71% and 88.72%, respectively, by germ-free BSFL and non-sterile BSFL for 10 days. Twenty-five AFB_1_-degrading bacteria were isolated from BSFL gut, and *S. acidaminiphila* A2 achieved the highest AFB_1_ degradation, by 94%. The exposure to AFB_1_ had an adverse effect on the growth performance of BSFL, resulting in a decrease of larvae survival rate and body weight. The supplementation of *S. acidaminiphila* A2 to BSFL could achieve complete AFB_1_ degradation in peanut meal and alleviate the negative effect of AFB_1_ on the growth performance of BSFL. Thus, it is promising to degrade AFB_1_ in contaminated feedstuff by BSFL in combination with commensal AFB_1_-degrading microorganisms.

## Figures and Tables

**Table 1:** Comparison of AFB_1_ degradation in peanut meal by germ-free BSFL and non-sterile BSFL (*n* = 3).

Items	Group ^1^		SEM ^2^	*p*-Valve
	GF-BSFL	NS-BSFL		
Dried frass weight (g)	8.29 ^a^	5.96 ^b^	0.09	<0.001
AFB_1_ concentration in frass (ng g^−1^)	74.13 ^a^	17.24 ^b^	2.09	<0.001
Total residual AFB_1_ content in frass (ng)	614.63 ^a^	101.53 ^b^	14.71	<0.001
AFB_1_ degradation rate (%)	31.71 ^a^	88.72 ^b^	1.64	<0.001

^a,b^ Values in the same row with no common superscript differ significantly (*p* < 0.05). ^1^ GF-BSFL, germ-free BSFL; NS-BSFL, non-sterile BSFL. ^2^ SEM, pooled standard error of the mean.

**Table 2:** Nutrient compositions of BSFL(*n* = 3).

Items	Group ^1^			SEM ^2^	*p*-Valve
	CON	AFB_1_	AFB_1_ + A2		
Crude fat (%)	23.49 ^b^	25.47 ^a^	25.53 ^a^	0.035	<0.001
Crude fiber (%)	8.90	8.90	9.00	0.019	0.125
Crude protein (%)	51.86 ^a^	51.46 ^ab^	51.03 ^b^	0.071	0.009
Aspartic acid (%)	3.90	3.94	3.81	0.029	0.253
Glutamate (%)	4.17	4.10	4.04	0.030	0.252
Serine (%)	1.25 ^a^	1.26 ^a^	1.20 ^b^	0.007	0.028
Arginine (%)	1.56	1.58	1.58	0.014	0.720
Glycine (%)	3.09	3.00	2.94	0.020	0.060
Threonine (%)	1.53	1.50	1.47	0.011	0.196
Prolin (%)	2.77 ^a^	2.65 ^b^	2.60 ^b^	0.018	0.025
Alanine (%)	4.25 ^a^	4.04 ^b^	3.94 ^b^	0.027	0.008
Valine (%)	2.68	2.61	2.56	0.018	0.087
Methionine (%)	0.65	0.62	0.61	0.007	0.124
Cystine (%)	0.31 ^b^	0.34 ^a^	0.30 ^b^	0.001	<0.001
Isoleucine (%)	1.98	1.96	1.90	0.013	0.086
Leucine (%)	2.90	2.88	2.78	0.020	0.093
Phenylalanine (%)	1.93	1.93	1.91	0.016	0.762
Histidine (%)	1.11	1.12	1.06	0.012	0.173
Lysine (%)	2.62	2.70	2.59	0.015	0.065
Tyrosine (%)	2.50	2.41	2.41	0.016	0.116

^a,b^ Values in the same row with no common superscript differ significantly (*p* < 0.05). ^1^ CON, non-sterile BSFL reared on peanut meal without AFB_1_; AFB_1_, non-sterile BSFL reared on peanut meal with AFB_1_; AFB_1_+A2, non-sterile BSFL reared on AFB_1_ contaminated peanut meal in the presence of *S. acidaminiphila* A2. ^2^ SEM, pooled standard error of the mean.

## Data Availability

The data presented in this study are available on request from the corresponding author.

## Supplementary Materials

The following supporting information can be downloaded at: https://www.mdpi.com/article/10.3390/life13010234/s1, Table S1: Identification of strains capable of degrading more than 60% of AFB_1_ by 16S rRNA gene analysis.

## References

[1] Reddy K.R.N., Salleh B., Saad B., Abbas H.K., Abel C.A., Shier W.T. (2010). An overview of mycotoxin contamination in foods and its implications for human health. Toxin Rev..

[2] My A., Ds S. (1997). Dietary factors affecting aflatoxin Bi carcinogenicity. Malays. J. Nutr..

[3] Gruber-Dorninger C., Jenkins T., Schatzmayr G. (2019). Global mycotoxin occurrence in feed: A ten-year survey. Toxins.

[4] Pandey M.K., Kumar R., Pandey A.K., Soni P., Gangurde S.S., Sudini H.K., Fountain J.C., Liao B., Desmae H., Okori P. (2019). Mitigating aflatoxin contamination in groundnut through a combination of genetic resistance and post-harvest management practices. Toxins.

[5] Bbosa G.S., Kitya D., Odda J., Ogwal-Okeng J. (2013). Aflatoxins metabolism, effects on epigenetic mechanisms and their role in carcinogenesis. Health.

[6] Ostry V., Malir F., Toman J., Grosse Y. (2017). Mycotoxins as human carcinogens-the IARC Monographs classification. Mycotoxin Res..

[7] Liu Y., Wu F. (2010). Global burden of aflatoxin-induced hepatocellular carcinoma: A risk assessment. Environ. Health Perspect..

[8] Rushing B.R., Selim M.I. (2019). Aflatoxin B_1_: A review on metabolism, toxicity, occurrence in good, occupational exposure, and detoxification methods. Food Chem. Toxicol..

[9] Guo Y., Zhao L., Ma Q., Ji C. (2021). Novel strategies for degradation of aflatoxins in food and feed: A review. Food Res. Int..

[10] Samuel M.S., Sivaramakrishna A., Mehta A. (2014). Degradation and detoxification of aflatoxin B_1_ by *Pseudomonas putida*. Int. Biodeterior. Biodegrad..

[11] Eshelli M., Harvey L., Edrada-Ebel R., McNeil B. (2015). Metabolomics of the bio-degradation process of aflatoxin B1 by *Actinomycetes* at an initial pH of 6.0. Toxins.

[12] Cserháti M., Kriszt B., Krifaton C., Szoboszlay S., Háhn J., Tóth S., Nagy I., Kukolya J. (2013). Mycotoxin-degradation profile of *Rhodococcus* strains. Int. J. Food Microbiol..

[13] Gao X., Ma Q., Zhao L., Lei Y., Shan Y., Ji C. (2011). Isolation of *Bacillus subtilis*: Screening for aflatoxins B_1_, M_1_, and G_1_ detoxification. Eur. Food Res. Technol..

[14] Allegretti G., Talamini E., Schmidt V., Bogorni P.C., Ortega E. (2018). Insect as feed: An emergy assessment of insect meal as a sustainable protein source for the brazilian poultry industry. J. Clean. Prod..

[15] Salam M., Shahzadi A., Zheng H., Alam F., Nabi G., Dezhi S., Ullah W., Ammara S., Ali N., Bilal M. (2022). Effect of different environmental conditions on the growth and development of black soldier fly larvae and its utilization in solid waste management and pollution mitigation. Environ. Technol. Innov..

[16] Sánchez-Muros María J., Barroso F.G., Manzano-Agugliaro F. (2014). Insect meal as renewable source of food for animal feeding: A review. J. Clean. Prod..

[17] Scala A., Cammack J.A., Salvia R., Scieuzo C., Franco A., Bufo S.A., Tomberlin J.K., Falabella P. (2020). Rearing substrate impacts growth and macronutrient composition of *Hermetia illucens* (L.) (Diptera: Stratiomyidae) larvae produced at an industrial scale. Sci. Rep..

[18] Schiavone A., Dabbou S., De Marco M., Cullere M., Biasato I., Biasibetti E., Capucchio M.T., Bergagna S., Dezzutto D., Meneguz M. (2018). Black soldier fly larva fat inclusion in finisher broiler chicken diet as an alternative fat source. Animal.

[19] Belghit I., Liland N.S., Waagbø R., Biancarosa I., Pelusio N., Li Y., Krogdahl Å., Lock E.-J. (2018). Potential of insect-based diets for Atlantic salmon (*Salmo salar*). Aquaculture.

[20] Bosch G., Van Der Fels-Klerx H.J., De Rijk T.C., Oonincx D.G.A.B. (2017). Aflatoxin B_1_ tolerance and accumulation in black soldier fly larvae (*Hermetia illucens*) and yellow mealworms (*Tenebrio molitor*). Toxins.

[21] Camenzuli L., Van Dam R., de Rijk T., Andriessen R., Van Schelt J., Van der Fels-Klerx H.J.I. (2018). Tolerance and excretion of the mycotoxins aflatoxin B_1_, zearalenone, deoxynivalenol, and ochratoxin a by *Alphitobius diaperinus* and *Hermetia illucens* from contaminated substrates. Toxins.

[22] Pei Y., Zhao S., Chen X., Zhang J., Ni H., Sun M., Lin H., Liu X., Chen H., Yang S. (2022). *Bacillus Velezensis* EEAM 10b strengthens nutrient metabolic process in black soldier fly larvae (*Hermetia illucens*) via changing gut microbiome and metabolic pathways. Front. Nutr..

[23] (2016). The Ministry of Health of the People’s Republic of China. National Food Safety Standard: Determination of Protein in Foods.

[24] (2016). The Ministry of Health of the People’s Republic of China. National Food Safety Standard: Determination of Fat in Foods.

[25] (2016). The Ministry of Health of the People’s Republic of China. National Food Safety Standard: Determination of Ash in Foods.

[26] (2016). The Ministry of Health of the People’s Republic of China. National Food Safety Standard: Determination of Amino Acids in Foods.

[27] Purschke B., Scheibelberger R., Axmann S., Adler A., Jäger H. (2017). Impact of substrate contamination with mycotoxins, heavy metals and pesticides on the growth performance and composition of black soldier fly larvae (*Hermetia illucens*) for use in the feed and food value chain. Food Addit. Contam. A.

[28] Meijer N., Stoopen G., Van der Fels-Klerx H.J., Van Loon J.J.A., Carney J., Bosch G. (2019). Aflatoxin B_1_ conversion by black soldier fly (*Hermetia illucens*) larval enzyme extracts. Toxins.

[29] Wu N., Wang X., Xu X., Cai R., Xie S. (2020). Effects of heavy metals on the bioaccumulation, excretion and gut microbiome of black soldier fly larvae (Hermetia illucens). Ecotoxicol. Environ. Saf..

[30] Luo X., Yang Q., Lin Y., Tang Z., Tomberlin J.K., Liu W., Huang Y. (2022). Black soldier fly larvae effectively degrade lincomycin from pharmaceutical industry wastes. J. Environ. Manag..

[31] Yang C., Ma S., Li F., Zheng L., Tomberlin J.K., Yu Z., Zhang J., Yu C., Fan M., Cai M. (2022). Characteristics and mechanisms of ciprofloxacin degradation by black soldier fly larvae combined with associated intestinal microorganisms. Sci. Total Environ..

[32] Guan S., Ji C., Zhou T., Li J., Ma Q., Niu T. (2008). Aflatoxin B_1_ degradation by *Stenotrophomonas maltophilia* and other microbes selected using coumarin medium. Int. J. Mol. Sci..

[33] Cai M., Qian Y., Chen N., Ling T., Wang J., Jiang H., Wang X., Qi K., Zhou Y. (2020). Detoxification of aflatoxin B_1_ by *Stenotrophomonas* sp. CW117 and characterization the thermophilic degradation process. Environ. Pollut..

[34] Zhao L., Guan S., Gao X., Ma Q., Lei Y., Bai X., Ji C. (2011). Preparation, purification and characteristics of an aflatoxin degradation enzyme from *Myxococcus fulvus* ANSM068. J. Appl. Microbiol..

[35] Guo Y., Qin X., Tang Y., Ma Q., Zhang J., Zhao L. (2020). CotA laccase, a novel aflatoxin oxidase from *Bacillus licheniformis*, transforms aflatoxin B_1_ to aflatoxin Q_1_ and epi-aflatoxin Q_1_. Food Chem..

[36] Yan Y., Zhang X., Chen H., Huang W., Jiang H., Wang C., Xiao Z., Zhang Y., Xu J. (2022). Isolation and aflatoxin B_1_-degradation characteristics of a *Microbacterium proteolyticum* B204 Strain from bovine faeces. Toxins.

[37] Sangare L., Zhao Y., Folly Y.M.E., Chang J., Li J., Selvaraj J.N., Xing F., Zhou L., Wang Y., Liu Y. (2014). Aflatoxin B_1_ degradation by a *Pseudomonas* strain. Toxins.

[38] Yue X., Ren X., Fu J., Wei N., Altomare C., Haidukowski M., Logrieco A.F., Zhang Q., Li P. (2022). Characterization and mechanism of aflatoxin degradation by a novel strain of *Trichoderma reesei* CGMCC3.5218. Front. Microbiol..

[39] Zhao D., Xie H., Gao L., Zhang J., Li Y., Mao G., Zhang H., Wang F., Lam S.S., Song A. (2022). Detoxication and bioconversion of aflatoxin B_1_ by yellow mealworms (*Tenebrio molitor*): A sustainable approach for valuable larval protein production from contaminated grain. Ecotoxicol. Environ. Saf..

