# Supplementary material for: Characteristics of Aflatoxin B1 Degradation by Stenotrophomonas acidaminiphila and It’s Combination with Black Soldier Fly Larvae
Source: Life (Basel). 2023 Jan 14;13(1):234. doi: 10.3390/life13010234 (PMC9865385; doi:10.3390/life13010234)
Supplement: Supplementary file 1 [file life-13-00234-s001.zip › life-2135915-supplementary.pdf]

Table S1. Identification of strains capable of degrading more than 60% of AFB_1 by 16S rRNA gene analysis.

Strain no.	Highest identity sequences	Identity(%)	Query cover(%)	GenBank accession no.
A1	*Bacillus paramycoides*	99.79	100	MT611870.1
A3	*Providencia stuartii*	100	99	CP044076.1
A6	*Bacillus cereus*	99.65	100	MW559320.1
A10	*Bacillus tropicus*	99.86	99	ON000567.1
A12	*Providencia stuartii*	99.86	99	CP044076.1
A13	*Escherichia coli*	100	100	OP986071.1
A15	*Escherichia coli*	100	100	MF428963.1
A16	*Escherichia fergusonii*	99.93	100	OP217090.1
A21	*Escherichia coli*	100	100	OP762770.1
A23	*Klebsiella pneumoniae*	99.93	100	CP030320.1
